# CAR-T Cell Therapy in Hematological Malignancies: Current Opportunities and Challenges

**DOI:** 10.3389/fimmu.2022.927153

**Published:** 2022-06-10

**Authors:** Xiaomin Zhang, Lingling Zhu, Hui Zhang, Shanshan Chen, Yang Xiao

**Affiliations:** ^1^ Department of Hematology, Jinshazhou Hospital of Guangzhou University of Chinese Medicine, Guangzhou, China; ^2^ Institute of Clinical Medicine College, Guangzhou University of Chinese Medicine, Guangzhou, China; ^3^ Cancer Center, Integrated Hospital of Traditional Chinese Medicine, Southern Medical University, Guangzhou, China; ^4^ School of Medicine, Jishou University, Jishou, China; ^5^ Department of Hematology, Shenzhen Qianhai Shekou Pilot Free Trade Zone Hospital, Shenzhen, China

**Keywords:** CAR-T cell, hematological malignancies, CAR-T related toxicities, antigen escape, immunosuppressive tumor microenvironment, combinatorial therapy

## Abstract

Chimeric antigen receptor T (CAR-T) cell therapy represents a major breakthrough in cancer treatment, and it has achieved unprecedented success in hematological malignancies, especially in relapsed/refractory (R/R) B cell malignancies. At present, CD19 and BCMA are the most common targets in CAR-T cell therapy, and numerous novel therapeutic targets are being explored. However, the adverse events related to CAR-T cell therapy might be serious or even life-threatening, such as cytokine release syndrome (CRS), CAR-T-cell-related encephalopathy syndrome (CRES), infections, cytopenia, and CRS-related coagulopathy. In addition, due to antigen escape, the limited CAR-T cell persistence, and immunosuppressive tumor microenvironment, a considerable proportion of patients relapse after CAR-T cell therapy. Thus, in this review, we focus on the progress and challenges of CAR-T cell therapy in hematological malignancies, such as attractive therapeutic targets, CAR-T related toxicities, and resistance to CAR-T cell therapy, and provide some practical recommendations.

## Introduction

Traditionally, the treatment of hematological malignancies mainly includes chemotherapy, radiotherapy, and hematopoietic stem cell transplantation (HSCT). However, with advances in tumor immunology, immune targeted therapy, such as monoclonal antibodies, bispecific antibodies, antibody-drug conjugates, and chimeric antigen receptor T (CAR-T) cell therapy, has opened a new avenue for the treatment of malignancies. In particular, CAR-T cell therapy has revolutionized the treatment of hematological malignancies and achieved unprecedented responses in recent years, especially in relapsed/refractory (R/R) B-cell acute lymphocytic leukemia (B-ALL), non-Hodgkin lymphoma (NHL), and multiple myeloma (MM). At present, there are six CAR-T cell products approved by the US Food and Drug Administration (FDA) for the treatment of R/R B cell malignancies, including tisagenlecleucel (Kymriah; Novartis), axicabtagene ciloleucel (Yescarta; Gilead), brexucabtagene autoleucel (Tecartus; Gilead), lisocabtagene maraleucel (Breyanzi; Bristol Myers Squibb), idecabtagene vicleucel (Abecma; Bristol Myers Squibb and Bluebird Bio), and ciltacabtagene autoleucel (Carvykti; Legend and Janssen), which target the most common target antigens CD19 and B cell maturation antigen (BCMA) (**Table 1** landmark clinical trials). In addition, there are a considerable number of novel targets which are being explored.

**Table 1:** Landmark clinical trials of FDA-approved CAR-T cell products.

CAR-T products	Target	Company	Year	Clinical trial	Indications	Response	Toxicities (Grade 3/4)	Reference
**tisagenlecleucel**	CD19	Novartis	2017	ELIANA	R/R B-ALL	ORR 81%, CR 60%	CRS (46%), CRES (13%) cytopenia (61%)	Maude SL et al. (1)
			2018	JULIET	R/R DLBCL	ORR 52%, CR 40%, PR 12%	CRS (22%), CRES (12%) cytopenia (32%) infections (20%)	Schuster SJ et al. (2)
			2021	ELARA	R/R FL	ORR 86%, CR 69%	CRS (49%), CRES (37%) infections (5%)	Fowler NH et al. (3)
**axicabtagene ciloleucel**	CD19	Gilead	2017	ZUMA-1	R/R DLBCL, transformed FL, PMBCL, and HGBCL	ORR 82%, CR 54%	CRS (13%), CRES (28%), cytopenia (78%)	Locke FL et al. (4)
**brexucabtagene autoleucel**	CD19	Gilead	2020	ZUMA-2	R/R MCL	ORR 85%, CR 59%,	CRS (15%), CRES (31%), cytopenia(94%), infections (32%)	Wang M et al. (5)
**lisocabtagene maraleucel**	CD19	Bristol Myers Squibb	2021	TRANSCEND	R/R DLBCL, HGBCL, PMBCL, and FL grade 3B	ORR 73%, CR 53%	CRS (2%), CRES (10%), cytopenia (60%)	Abramson JS et al. (6)
**idecabtagene vicleucel**	BCMA	Bristol Myers Squibb and Bluebird Bio	2021	KarMMa	R/R MM	ORR 73%, CR 33%	CRS (5%), CRES (3%), cytopenia (89%)	Berdeja JG et al. (7)
**ciltacabtagene autoleucel**	BCMA	Legend and Janssen	2022	CARTITUDE-1	R/R MM	ORR97%, CR 67%	CRS (4%), CRES (9%), cytopenia (95%)	Munshi NC et al. (8)

However, despite the remarkable breakthrough of CAR-T therapy in B cell malignancies, severe toxicities associated with CAR-T cell therapy may compromise its efficacy and could even progress into life-threatening conditions, such as multiple organ dysfunction, sepsis, and disseminated intravascular coagulation (DIC). Cytokine release syndrome (CRS) is the most common complication, which is a systemic inflammatory response induced by the overactivation of CAR-T cells and endogenous immune cells, such as macrophages and dendritic cells. Its manifestations are diverse and partially similar with infections. Moreover, severe CRS is correlated with the increased risk of CAR-T-cell-related encephalopathy syndrome (CRES) and coagulopathy (9). Thus, clarifying their underlying mechanisms could facilitate the prevention and management of these adverse events. In addition, the resistance after CAR T-cell therapy cannot be ignored.

Thus, this review aims to introduce the advances and challenges in CAR-T cell therapy, such as attractive therapeutic targets, toxicities related to CAR-T cell therapy, and resistance to CAR-T cell therapy, and explore their underlying mechanisms and effective treatment strategies in order to facilitate the application and management of CAR-T cell therapy.

## Overview of CAR-T Cell Therapy

To manufacture CAR-T cells, T cells are collected from peripheral blood of patients or donors and then genetically engineered *in vitro* to express chimeric antigen receptor (CAR). Thus, CAR-T cells recognize specific surface antigens on tumor cells without antigen processing and presentation, which indicates that antigen recognition by CAR-T cells is independently of major histocompatibility complex (MHC) restriction. After genetic modifications, they undergoextensive expansion *in vitro*. Then the patients receive lymphodepleting chemotherapy to make room for these adoptive CAR-T cells, and subsequently these genetically engineered CAR-T cells are re-infused into the patients. These CAR-T cells specifically recognize target antigens and rapidly proliferate to exert anti-tumor effects *in vivo*.

**Figure 1:**
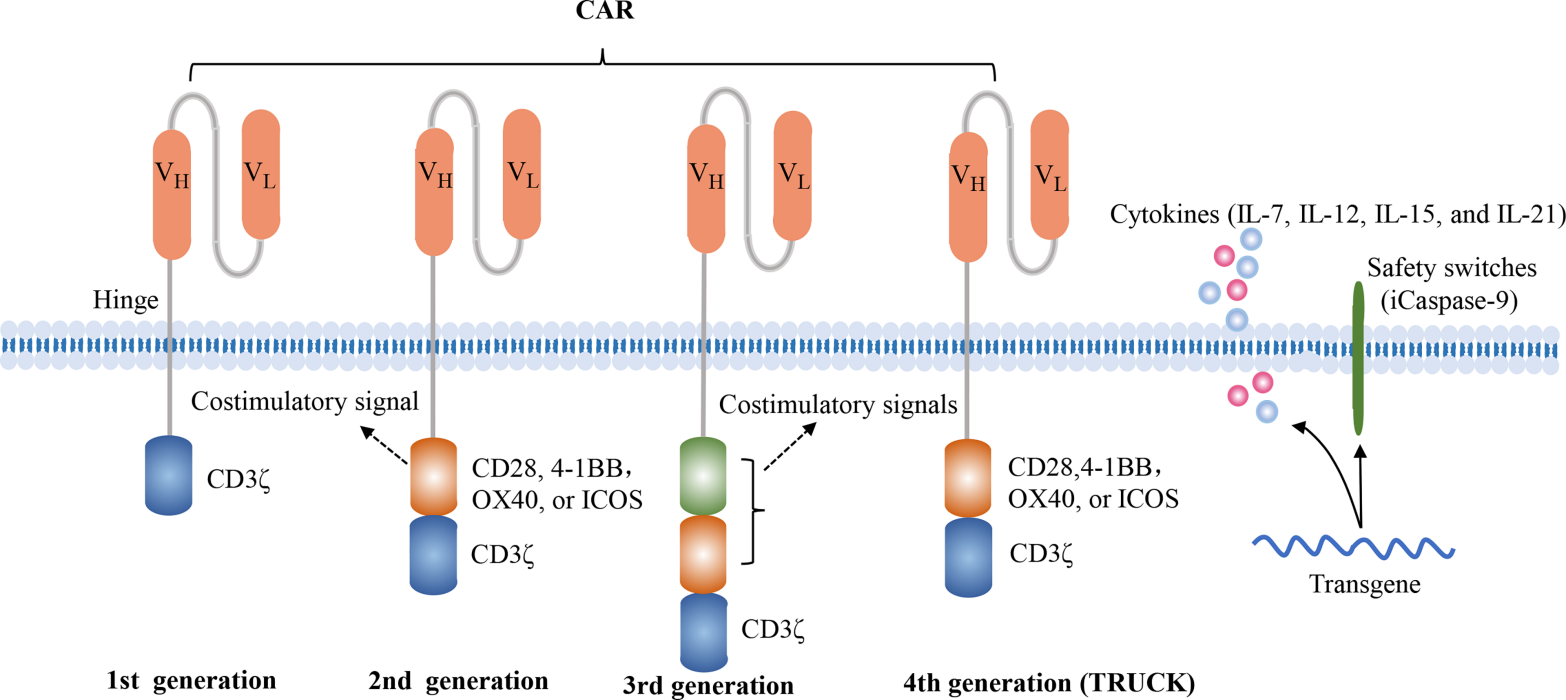
Four generations of CAR constructs. The first-generation of CAR consists of the antigen recognition domain scFv and an intracellular T cell activation domain CD3ζ. The second-generation CAR adds a costimulatory molecule, such as CD28, 4-1BB, OX40 or ICOS, which enables the T cells to obtain a superior proliferative capacity and secrete large amounts of cytokines. The third-generation CAR contains two distinct costimulatory domains, such as CD28 and 4-1BB. The fourth-generation of CAR, also known as TRUCK or armored CAR, is additionally equipped with safety switches or engineered to secrete cytokines in order to regulate the persistence or the function of CAR-T cells, such as iCaspase-9, IL-7, IL-15, IL-21.

The CAR structure consists of an extracellular antigen-recognition domain, a transmembrane domain, and an intracellular signaling domain. The extracellular domain, a single-chain variable fragment (scFv), is able to specifically recognize tumor surface antigens. Typically, tumor antigens are categorized into tumor-associated antigens (TAAs) and tumor specific antigens (TSAs), and most of they are TAAs. Once TAAs are identified by scFv, CAR-T cells are activated and transmit activation signals to the intracellular domain. The first-generation CAR construct contains an antigen-recognition domain scFv and an intracellular CD3ζ activation domain. Due to the absence of costimulatory signals, they exhibit the limited proliferative capacity and anti-tumor effects. The second-generation CAR construct adds a costimulatory domain, such as CD28, 4-1BB, OX40, or ICOS, which enables themselves to possess the better proliferative capacity and release more cytokines. Currently, these commercial CAR-T cell products both utilize the second-generation CAR construct. The third-generation CAR construct encompasses two distinct costimulatory molecules, such as CD28 and 4-1BB. The fourth-generation CAR construct, also named TRUCK or armored CAR, is additionally modified to secrete cytokines or express suicide genes, such as IL-7, IL-12, IL-15, IL-21, and iCaspase-9 (10, 11) (**Figure 1**). The fourth-generation CAR-T cells may be more effective in eliminating tumor cells by activating the endogenous immune responses. However, the characteristics of fourth-generation CAR-T cells are largely unknown. In addition, the 2020 American Society of Hematology (ASH) annual meeting announced two studies about FasT CAR-T cells, including CD19-CD22 FasT dual-targeting CAR-T cells (GC022F) in B-ALL patients and BCMA-CD19 FasT dual-targeting CAR-T cells (GC012F) in R/R MM patients (12, 13). These FasT CAR-T products were manufactured in 24 to 36 hours and showed superior efficacy in preliminary studies, indicating that they might be more suitable for rapidly progressive B cell malignancies.

At present, all commercial CAR-T cell products are manufactured using autologous T lymphocytes, but high manufacturing costs, relatively longer manufacturing cycle, and decreased number and function of lymphocytes after multiline chemotherapies have restricted their further application. However, it seems that universal CAR-T (UCAR-T) cells which are derived from healthy donors are able to overcome these limitations. The large-scale production of UCAR-T cells makes them “off-the-shelf” products, which could reduce manufacturing costs and increase their accessibility. Unfortunately, these allogeneic UCAR-T cells could induce graft versus host disease (GVHD) (14). Furthermore, the host immune system is able to reject these donor-derived UCAR-T cells and impairs their persistence. In addition, due to the excellent natural killing functions of NK cells and their abundant sources, such as NK92 cell line, cord blood, peripheral blood, and induced pluripotent stem cells, as well as no induction of GVHD, CAR-NK cells are currently being explored (15). 

## Attractive Targets for CAR-T Cell Therapy in Hematological Malignancies

**Figure 2:**
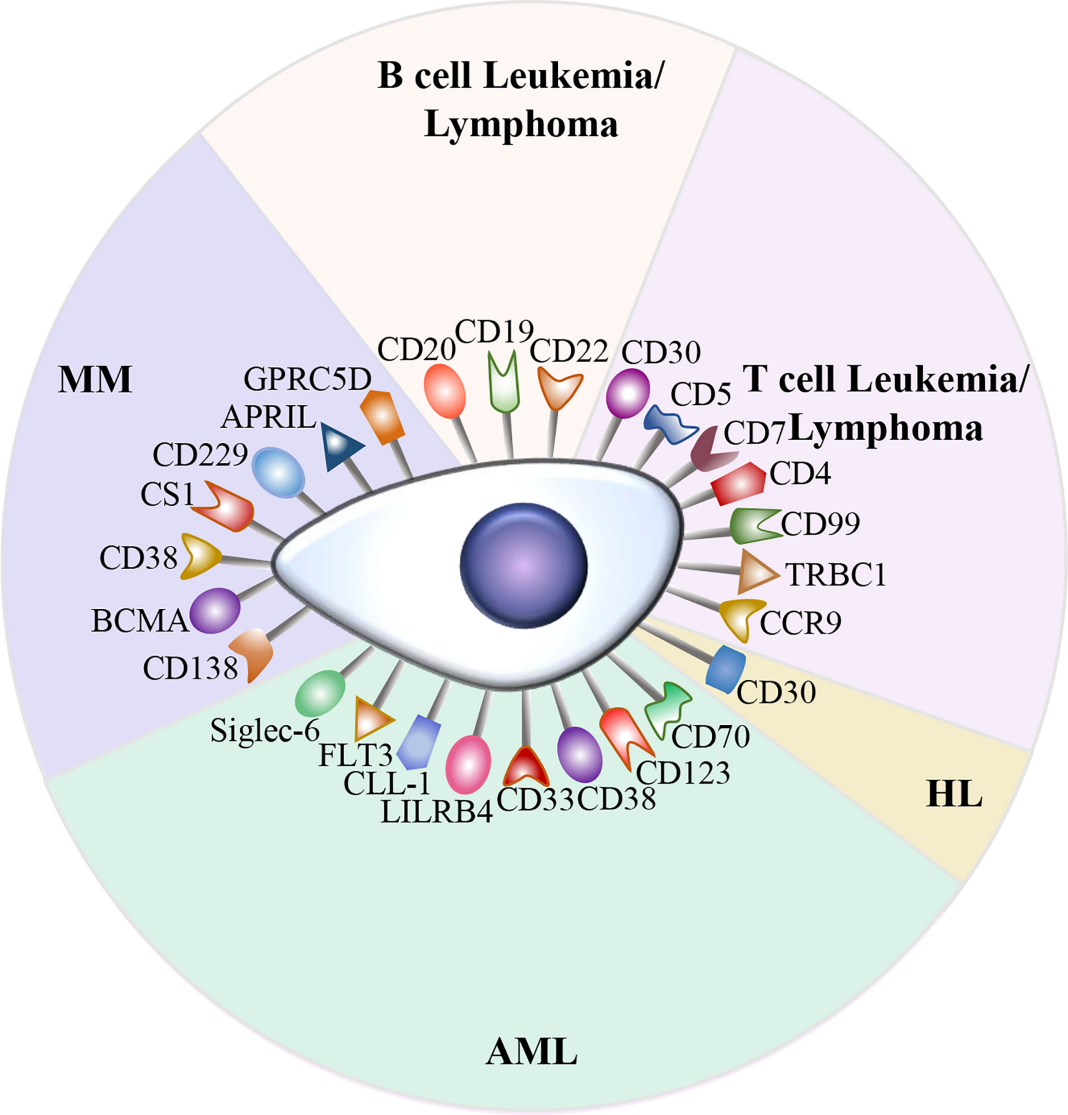
Potential therapeutic targets in hematological malignancies. A variety of attractive targets for CAR-T cell therapy in hematological malignancies, including T and B cell leukemia/lymphoma, HL, AML, and MM.

Currently, CD19 and BCMA are the most common targets in CAR-T cell therapy. Although anti-CD19 CAR-T cell therapy and anti-BCMA CAR-T cell therapy have achieved outstanding outcomes in B cell malignancies, relapse after CAR-T cell therapy is frequently observed. In addition, due to the antigenic heterogeneity of acute myeloid leukemia (AML) as well as the lack of CD19 expression in Hodgkin lymphoma (HL) and T cell malignancies, a number of potential targets are currently being investigated (**Figure 2**).

### Targets for CAR-T Cell Therapy in B Cell Lymphoblastic Leukemia/Lymphoma

The CD19 is one of the most important target antigens in B cell malignancies, including B-ALL and NHL. In recent years, anti-CD19 CAR-T cell therapy has achieved rapid and durable responses in patients with R/R B-ALL and NHL (1–6, 16), and has dramatically altered the therapeutic landscape of B cell malignancies. Until now, four anti-CD19 CAR-T cell products have been approved by FDA for the treatment of R/R B-ALL and NHL (17). Despite the outstanding clinical results of anti-CD19 CAR-T therapy, CD19 antigen loss is frequently observed (18). Thus, the alternative targets for CAR-T cell therapy in R/R B-ALL and NHL have been explored.

CD20 is overexpressed in over 90% of B cell lymphomas and identified as an attractive target for CD20 positive B cell lymphomas, and the anti-CD20 monoclonal antibody rituximab has showed the excellent effect on NHL over the past few years. In an early clinical trial, the overall response rate (ORR) of anti-CD20 CAR-T cell therapy in DLBCL patients was 86% (19). In subsequent phase 1 and 2 trials, the anti-CD20 CAR-T cells were administrated to 17 R/R NHL patients, and 54.5% of patients achieved complete remissions (CRs) and 12 patients remained in remission with a median follow-up of 20 months (20). To prevent antigen escape, the combination of anti-CD19 and anti-CD20 CAR-T cells for the treatment of R/R DLBCL was investigated, and this combinational therapy was demonstrated to be safe and feasible (21). CD22 is highly expressed on most B cell malignancies, including B-ALL and DLBCL (22, 23). In particular, it is restrictively expressed on normal B cells and not expressed on hematopoietic stem cells, so it is an ideal target for CAR-T cell therapy in R/R B-ALL and DLBCL. In several clinical trials, the anti-CD22 CAR-T cell therapy has shown excellent efficacy in R/R B-ALL and R/R DLBCL patients who have failed in previous anti-CD19 CAR-T cell therapy (24–26). In addition, the humanized anti-CD22 CAR- T cells exhibits potent activity against leukemia cells with low CD22 expression (27).

### Targets for CAR-T Cell Therapy in T Cell Lymphoblastic Leukemia/Lymphoma

Patients with R/R T-cell acute lymphoblastic leukemia (T-ALL) and T cell lymphomas often have poor prognosis. Compared with the outstanding clinical outcomes of anti-CD19 CAR-T cell therapy in B cell malignancies, the efficacy and safety CAR-T cell therapy in T cell malignancies are largely unknown and under investigation. CD7 is highly expressed in 95% of T-ALL patients and represents a desirable target for the treatment of T-ALL (28). In an open-label and single-arm clinical trial, 2 R/R T-ALL patients were treated with allogeneic anti-CD7 CAR-T cell therapy. One patient remained in remission for over 1 year, while the other relapsed 48 days after CAR-T cell infusion (28). In another phase I clinical trial, 20 R/R T-ALL patients received donor-derived anti-CD7 CAR-T cell infusion, and 90% of participants achieved CRs (29). In addition, a case study reported that an 11-year-old T-ALL patient who didn’t respond to induction failure was treated with autologous anti-CD7 CAR-T cell therapy, and he achieved remission on day 17 and subsequently underwent HSCT (30). CD5 is expressed in approximately 85% of T cell malignancies, such as T-cell lymphoblastic lymphoma (T-LBL) and peripheral T-cell lymphoma (PTCL). A recent study has demonstrated that anti-CD5 CAR-T cells effectively eliminated malignant T cells (31). In a phase I clinical trial, a refractory T-LBL patient with central nervous system (CNS) infiltration received the anti-CD5 CAR-T cell therapy and achieved CR within 4 weeks (32). Additionally, anti-CD4 CAR-T cells showed superior activity against T cell malignancies in preclinical studies (33). However, all three targets CD4, CD5 and CD7 are expressed on normal T cells, so targeting them may result in the depletion of normal T cells and the fratricide of CAR-T cells (34). Malignant T cells express T cell receptor β-chain constant domains 1 (TRBC1), so anti-TRBC1 CAR-T cells are able to selectively eliminate TRBC1 positive malignant T cells. Importantly, it could retain a majority of normal T cells *in vivo* (35). CD99 is highly expressed in newly diagnosed T-ALL patients, and it represents a novel target for T-ALL (36). Furthermore, chemokine receptor CCR9 is expressed in over 70% of T-ALL patients, and only on less than 5% of normal T cells. In addition, it is correlated with multidrug resistance and poor prognosis. Thus, it represents an ideal target for CCR9 positive T-ALL. In preclinical studies, anti-CCR9 CAR-T cells exhibited potent anti-leukemic activity and were resistant to fratricide (37).

In addition, CD30 is highly expressed in anaplastic large-cell lymphoma (ALCL) and is variably expressed in PTCL subtypes. In particular, CD30 is restrictively expressed on normal T cells. Thus, CD30 is an ideal target for these lymphoma subtypes, and the anti-CD30 antibody-drug conjugate brentuximab vedotin (BV) has shown a high response rate in newly diagnosed PTCL patients (38, 39). Given the encouraging clinical efficacy of BV, the anti-CD30 CAR-T cells were developed and exhibited remarkable cytotoxicity against CD30 positive lymphomas in preclinical studies (40, 41).

### Targets for CAR-T Cell Therapy in Hodgkin Lymphoma

Anti-CD19 CAR-T cell therapy has shown excellent results in R/R B cell NHL. However, HL lacks the expression of CD19. Interestingly, CD30 is universally expressed in classical HL. Currently, several clinical trials have been carried out to evaluate the safety and efficacy of anti-CD30 CAR-T cell therapy in R/R HL (42–45). In a clinical trial from China, 5 of 6 HL patients achieved CRs after the infusion of the third-generation anti-CD30 CAR-T cells, and the long-term remission lasted over 24 months in 3 patients (45). In another phase 1/2 clinical trial, 27 patients were treated with the anti-CD30 CAR-T cells and 67% of patients achieved CRs within 6 weeks, but 63% of patients experienced disease progression with a median follow-up of 9.5 months (46). In addition, the expression of CD30 in HL was down-regulated after anti-CD30 CAR-T cell therapy (47).

### Targets for CAR-T Cell Therapy in Acute Myeloid Leukemia

AML is the most common acute adult leukemia. Unfortunately, due to antigenic heterogeneity, the CAR-T cell therapy in AML has not achieved the same success as ALL. Recently, CD123, CD33, CD38, CD70, C-type lectin-like molecule-1(CLL-1), leukocyte immunoglobulin-like receptor-B4 (LILRB4), FMS-like tyrosine kinase 3 (FLT3) and sialic acid-binding immunoglobulin-like lectin 6 (Siglec-6) have been explored. CD123 and CD33 are highly expressed on leukemic stem cells in over 80% of AML patients, but they are expressed on hematopoietic stem cells as well (48). Accordingly, targeting them could increase the risk of long-term myelosuppression (49). To decrease hematological toxicity, a rapidly switchable universal anti-CD123 CAR-T cells were prepared (50, 51). In a small study, 3 R/R AML patients were treated with this universal anti-CD123 CAR-T cells, and all of them achieved a clinical response with the rapid hematologic recovery after the withdrawal of switch-mediated co-stimulation (52). In another phase 1 trial, 3 R/R AML patients received autologous anti-CD33 CAR-T cell infusion. Unfortunately, all of them died from disease progression (53). CD38 is expressed on most AML blast cells, and anti-CD38 CAR-T cell therapy was demonstrated to be effective in relapsed AML after allogeneic HSCT (54). CLL-1, which has been identified as an myeloid cell surface marker, is overexpressed on leukemic stem cells (55). Importantly, it is absent on hematopoietic stem cells. The CLL-1-targeted CAR-T cells specifically eliminated CLL-1 positive leukemia in preclinical studies (56, 57). CD70 is expressed on AML blasts but not on normal myeloid cells, making it a promising target for the treatment of AML (58, 59). Currently, the safety and efficacy of anti-CD70 CAR-T cell therapy are under investigation. In addition, LILRB4 is highly expressed on monocytic AML cells, and it is an attractive target for monocytic AML (60). Siglec-6 is expressed in approximately 60% of AML patients and absent on normal hematopoietic stem and progenitor cells. In preclinical studies, Siglec-6 CAR T cells effectively eliminated AML blasts in an AML mouse xenotransplantation model (61). Thus, it could serve as a well-validated target for CAR-T cell therapy in AML. In addition, nucleophosmin 1 (NPM1) mutations have been observed in 30%-35% of AML patients, and they are considered to be initiating mutations in leukemic cells. In preclinical mouse models, CAR-T cells targeting a nucleophosmin neoepitope which is presented by HLA-A2 exhibited potent specific anti-leukemia activity (62).

FLT3 is a transmembrane tyrosine kinase expressed on malignant blasts in approximately 30% of AML patients. FLT3 mutations include point mutations and an internal tandem duplication (ITD), and FLT3‐ITD is correlated with poor prognosis. In preclinical studies, the FLT3-targeted CAR-T cells successfully eliminated FLT3 positive AML cells (63, 64), and the FLT3 inhibitor crenolanib promoted their anti-tumor effects (64). Unfortunately, FLT3 is also expressed on normal hematopoietic stem and progenitor cells, so the FLT3-targeted CAR-T cells may affect normal hematopoiesis (64, 65).

### Targets for CAR-T Cell Therapy in Multiple Myeloma

MM remains an incurable plasma cell malignancy. With the application of novel agents, such as proteasome inhibitors, immunomodulatory drugs, and anti-CD38 monoclonal antibodies, MM patients have significantly improved survival outcomes (66). However, almost all MM patients inevitably relapse. BCMA is highly selectively expressed on malignant plasma cells, so it represents one of the most promising therapeutic targets for MM. At present, anti-BCMA CAR-T cell therapy has been demonstrated to be effective in R/R MM and achieved unprecedented responses (7, 8, 67–69), and two anti-BCMA CAR-T cell products, idecabtagene vicleucel and ciltacabtagene autoleucel, have been approved by the FDA for the treatment of R/R MM (7, 70). Furthermore, the anti-BCMA CAR-T cell therapy is effective in R/R MM patients with extramedullary disease (71–73). However, some MM patients still relapse after anti-BCMA CAR-T therapy, and BCMA expression is downregulated under therapeutic pressure. Therefore, new target antigens are required (74).

Currently, several potential target antigens have been investigated, such as CD38, CD138, CD229, SLAMF7, a proliferation-inducing ligand (APRIL), and G protein-coupled receptor, class C group 5 member D (GPRC5D). CD138 is highly expressed on MM cells and promote their survival and proliferation. In a preclinical study, anti-CD138 CAR-T cells effectively eliminated MM cells (75). In a small clinical trial, 5 patients received anti-CD138 CAR-T cell therapy, and 4 of them had a clinical response and remained stable for at least three months (76). CD38 is not only highly expressed on MM cells, but also expressed on hematopoietic cells and activated lymphocytes cells (77). Unfortunately, although the anti-CD38 CAR-T cells exhibited significant anti-tumor effects in mouse models, they impaired normal hematopoietic cells and lymphocytes (78). Clinically, CD38 is frequently combined with other targets, such as BCMA and CD138, to produce bispecific CAR-T cells, thereby reducing the risk of antigen escape (79, 80). CD229 is a surface antigen highly expressed on MM cells (81). The anti-CD229 CAR-T cells effectively eliminated MM cells in preclinical studies (82). SLAMF7, also known as CS1, is highly expressed in over 95% of MM patients. Similar to CD38, SLAMF7 is also expressed on normal lymphocytes, including activated T cells, NK cells, and B cells (83). Thus, SLAMF7 CAR-T cells could kill normal lymphocytes and increase the risk of CAR-T cell fratricide (84). Currently, the clinical trial of SLAMF7 CAR-T cells is ongoing (85). APRIL is a natural ligand which could directly bind to BCMA and transmembrane activator and CAML interactor (TACI). Thus, APRIL-targeted CAR-T cells recognize both BCMA and TACI expressed on MM cells, which may decrease the risk of antigen escape (86), and preserving its trimeric conformation could improve the anti-tumor activities (87). In addition, TGPRC5D is expressed on more than 50% of CD138 positive malignant plasma cells in bone marrow of MM patients, which also represents a potential target for the treatment of MM (88).

## Toxicities Related to CAR-T Cell Therapy and Their Underlying Mechanisms

Although CAR-T cell therapy has achieved great success in hematological malignancies, the adverse events related to CAR-T cell therapy remain to be a major challenge, such as CRS, CRES, B cell aplasia, cytopenia, and CRS-related coagulopathy. Without active and effective interventions, these complications might be life-threatening. In order to effectively manage these complications, it is important to explore their underlying mechanisms and recognize them in early stages.

### Cytokine Release Syndrome

CRS is one of the most common toxicities of CAR-T cell therapy. The incidence of CRS depends on a variety of factors, including disease characteristics, CAR structure, tumor burden, and CAR-T cell doses (89). The clinical manifestations of CRS are diverse, but they are frequently characterized by fever, fatigue, myalgia, poor appetite, hypoxia, hypotension, and even organ dysfunction. If left untreated, it might rapidly progress into life-threatening conditions, such as hemodynamic instability and multiple organ dysfunction. However, the recent study revealed that the patients with ≥ grade 2 CRS had higher rates of remission and longer progression-free survival (PFS) compared with those with < grade 2 CRS, which indicates that appropriate CRS might facilitate the efficacy of CAR-T therapy (90). Because CRS are primarily mediated by IL-6, IL-6 receptor antagonist tocilizumab is mainly recommended to relieve the clinical symptoms of CRS. According to different CRS grading, different treatment regimens are adopted. The symptomatic treatment and the supportive treatment are indicated for grade 1 CRS. Tocilizumab and corticosteroids are recommended for grade 3 and 4 CRS as well as grade 2 CRS accompanied by severe symptoms. Moreover, IL-1 is another important cytokine involved in CRS and CRES, and IL-1 receptor antagonist anakinra has been demonstrated to ameliorate both CRS and CRES (91–95). Furthermore, GM-CSF deficiency or inhibition not only can alleviate CRS and CRES by inhibiting the local infiltration of myeloid cells and T cells, but also enhance the anti-tumor effects of CAR-T cells (96, 97). Besides, the severity of CRS is positively associated with the patient’s tumor burden (89). To reduce tumor burden, traditional chemotherapy and radiotherapy could serve as the effective bridging strategies before CAR-T cell infusion.

**Figure 3:**
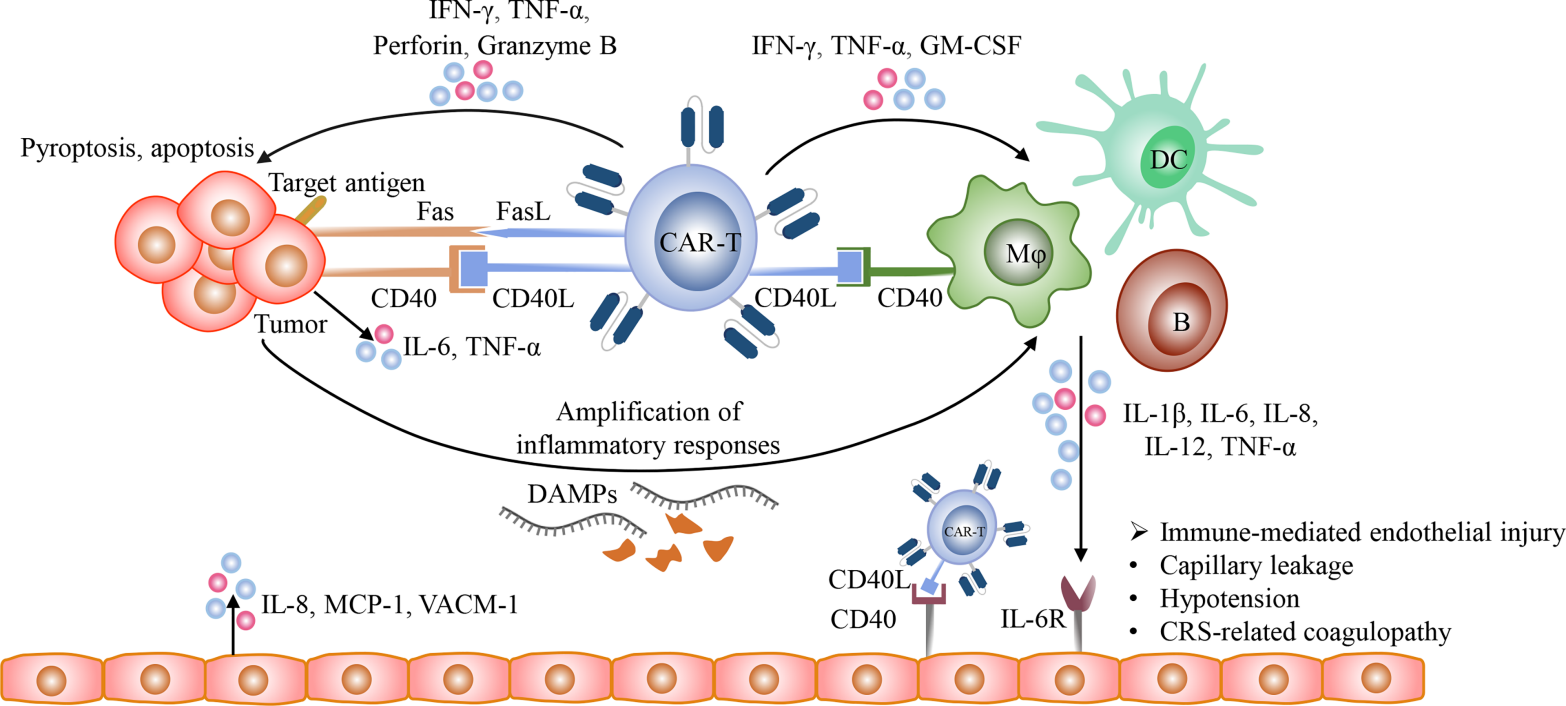
The mechanisms of CRS. After the recognition of target antigens, CAR T-cells rapidly proliferate and release multiple cytotoxic molecules, such as granzyme, perforin, IFN-γ, and TNF-α, and upregulate the expression of CD40L and Fas ligand (FasL), and eventually induce pyroptosis and apoptosis of tumor cells. Besides, the CD40/CD40L interactions between tumor cells and CAR T-cells promote Fas-mediated apoptosis. Then the lysed tumor cells release large amounts of DAMPs, such as HMGB1, which could activate innate immune cells, including macrophages and dendritic cells, further amplifying inflammatory responses. In addition, the CD40/CD40L interactions participate in the activation of various immune cells, including T cells, B cells, macrophages, dendritic cells, and conditional innate immune cells such as endothelial cells. The activated CAR-T cells with the increased expression of CD40L could activate macrophages and endothelial cells and promote their production of pro-inflammatory cytokines in a CD40-dependent manner. The cytokines released from activated immune cells could bind to their receptors on endothelial cells and then mediate endothelial dysfunction, resulting in capillary leakage and the release of procoagulant factors.

The detailed mechanisms of CRS remain incompletely understood. After recognizing target antigens, CAR-T cells are rapidly activated and secrete a large amount of granzyme, perforin, IFN-γ, and TNF-α. Perforin forms pores on tumor cell membrane and allows granzyme B to enter tumor cells. Granzyme B activates GSDME which is widely expressed on CD19 positive malignant B cells, resulting in tumor cell pyroptosis and the release of danger associated molecular patterns (DAMPs), such as high-mobility group box 1 (HMGB1) (98–101). Then DAMPs recruit and activate endogenous innate immune cells, such as macrophages and dendritic cells, thereby amplifying inflammatory responses and increasing the release of cytokines, including IL-1β and IL-6. Currently, it has been demonstrated that macrophages and monocytes rather than CAR-T cells are the major sources of these cytokines and contribute to CRS (91, 92, 102, 103). In addition, the CD40 and CD40 ligand (CD40L) interactions between CAR-T cells and host antigen-presenting cells (APCs) as well as tumor cells also play an important role in immune activation and the release of cytokines (104–106). CD40 is expressed on multiple APCs, including B cells, macrophages, dendritic cells (DCs), and monocytes, and highly expressed in a variety of hematological malignancies, such as NHL, AML, MM (105–108). CD40L is highly expressed on activated T cells, including CAR-T cells (104, 109). The contact-dependent CD40/CD40L interactions enhance the antigen presentation of APCs and CD40 positive malignancies and promote their secretion of cytokines, such as IL-1β, IL-6, and TNF-α (110, 111) (**Figure 3**). In addition, because endothelial cells act as conditional innate immune cells and express CD40, the CD40/CD40L interactions also participate in the activation of endothelial cells.

Since lymphoma lesions are mostly localized, CRS in lymphoma exhibits some distinct manifestations, such as local CRS, which is characterized by local redness, swelling, and heat. Then, CAR-T cells persistently expand and overflow into the circulation, and experience redistribution as well as mediating tissue damage. Finally, inflammation subsides and the impaired tissues are gradually repaired (112). Thus, the early management of local CRS is helpful to reduce the occurrence of subsequent systemic CRS.

### CAR-T-Cell-Related Encephalopathy Syndrome

CRES, also known as CAR-T cell-related neurotoxicity, is another common toxicity during CAR-T cell therapy. It usually occurs simultaneously with CRS or later than CRS. The manifestations of CRES include headache, dizziness, delirium, seizures and cerebral edema. Due to the lack of suitable animal models, the underlying pathological mechanisms of CRES are not fully understood. Severe CRS, high tumor burden, and excessive CAR-T cell expansion might be correlated with the increased risk of CRES. Currently, immune-mediated endothelial activation is a well-established mechanism involved in the occurrence of CRES (113–115). Upon the recognition of target antigens, CAR-T cells rapidly expand and secrete cytokines to activate endogenous immune cells, such as macrophages, which in turn release large amounts of cytokines and activate cerebral microvascular endothelial cells, eventually resulting in the disruption of tight junctions and the increased blood-brain barrier (BBB) permeability (114, 115). Then, the high concentrations of serum cytokines enter the BBB by passive diffusion, and the elevated levels of pro-inflammatory cytokines in CSF seem to be associated with CRES, including IL-1β, IL6, IL-8, IFN-γ, GM-CSF, MCP-1, and granzyme B (116–118). In addition, it has been demonstrated that T cells and macrophages, including CAR-T cells, could infiltrate into the CNS due to the disruption of the BBB (118–121). These infiltrated immune cells and cytokines could induce the activation of microglia which are brain-resident macrophages, further amplifying local inflammatory responses and eventually resulting in neurotoxicity (115, 119, 122–124). Thus, immune-mediated endothelial injury is a trigger factor for CRES (113–115, 125). As tocilizumab couldn’t cross the BBB, it exhibits the limited efficacy in the management of CRES. Given the increased CNS penetration of corticosteroids, it is recommended for the treatment of CRES, and it could not affect the proliferation and the anti-tumor effects of CAR-T cells (126).

### B/T Cell Aplasia and Infections

CD19 and CD20 are expressed on multiple differentiated B-lineage cells as well as malignant B cells, and represent attractive targets for CAR-T cell therapy in B-ALL and lymphoma. BCMA, a plasma cell-selective protein, is highly expressed on MM cells as well as mature B cells and normal plasma cells. Thus, CD19‐targeted, CD20‐targeted and BCMA‐targeted CAR T cells exhibit superior anti-tumor activity in B cell malignancies (1, 3, 67, 127), but they attack normal B cells as well, which could result in impaired humoral immunity, such as B cell aplasia and hypogammaglobulinemia (128–131). Moreover, lymphodepleting chemotherapy prior to CAR-T cell infusion could also impair host immunity. Due to impaired host immunity, these individuals are more susceptible to infections (132). It has been demonstrated that most of infection events occur during the first 30 days of CAR-T cell infusion, and the bacterial infection predominates, mainly including bloodstream infection and respiratory infection (133). In a phase 1/2 study, 31% of the patients who received the anti-CD19 CAR-T therapy experienced infections between day 31 and day 180 (134). Thus, the long term follow-up and the detection of gamma globulin levels might be helpful. To restore humoral immunity, immunoglobulin supplementation is essential for these immune-compromised individuals. In addition, the high-dose CAR-T cell infusion seems to be associated with the infections after CAR-T cell infusion (134, 135). Thus, CAR-T cells can be administrated in a dose-escalation regimen. Furthermore, CAR-T cell therapy increases the risk of HBV reactivation in patients with resolved HBV infections due to persistent B-cell aplasia, so antiviral prophylaxis and regular monitoring of the virus are recommended (128, 136, 137). Unfortunately, with the application of CAR-T cell therapy in T cell malignancies, T cell aplasia might be observed because a majority of target antigens are co-expressed on normal T cells (34).

However, it is difficult to differentiate between CRS and infections due to the similar clinical manifestations, such as pyrexia and the elevated levels of pro-inflammatory cytokines and C-reactive protein (CRP). Moreover, CRS is likely to occur simultaneously with infections. In order to avoid the life-threatening infections during CAR-T cell therapy, the early recognition and management of infections is important. Nevertheless, it is usually not timely to identify the infection by blood culture (133). Thus, the detection of special biomarkers or the establishment of a prediction model for infection is critical. IL-6 is one of the key cytokines involved in the infection-induced cytokine storm and CAR-T cell therapy-induced CRS. Typically, the elevation of serum IL-6 associated with CRS occurs within 3 weeks after CAR-T cell infusion, so the “double peaks of IL-6” is identified as one of the characteristics of life-threatening infections. Compared with blood culture, it seems that employing the “double peaks of IL-6” pattern to predict the life-threatening infection is faster (135). When infection is suspected, empiric anti-infective treatment should be initiated immediately once blood and sputum samples are collected for the detection of pathogenic microorganisms, especially in neutropenic patients. In addition, Herpesvirus infections have been occasionally observed in several clinical trials (138–140). To prevent herpesvirus infections, it’s recommended that acyclovir 400 mg should be prophylactically administrated twice daily from lymphodepletion chemotherapy to at least 6 months post CAR-T cell infusion (141).

### Cytopenia

Cytopenia is frequently observed during CAR-T cell therapy and lasts for several days to months, including anemia, thrombocytopenia, and leukopenia, and the incidence of cytopenia range from 30% to 100% in clinical trials (2, 29, 72, 79, 142, 143). It has been demonstrated that cytopenia is associated with severe CRS (144–146). Under inflammatory conditions, CD40 is significantly up-regulated on granulocytic progenitor/precursor cells which also express low levels of CD40L, and the CD40/CD40L interactions between granulocytic progenitor/precursor cells significantly promote their own apoptosis (147). In addition, the pro-inflammatory cytokines, such as IL-1, TNF-α, and HMGB1, could suppress erythropoietin production (148, 149), and the activated macrophages could destroy erythrocytes (150). The limited hematopoietic capacity mediated by prior chemotherapy and HSCT might be involved in cytopenia as well (151–153). Furthermore, some target antigens are co-expressed on normal hematopoietic stem or progenitor cells, so CAR-T cells could directly mediate the destruction of hematopoietic cells (48, 49). Clinically, red blood cell and platelet transfusions and hematopoietic growth factors, such as granulocyte colony-stimulating factor (G-CSF) and thrombopoietin (TPO), as well as TPO receptor agonists and sirolimus, are able to ameliorate cytopenia (154, 155).

### CRS-related Coagulopathy

As a newly identified toxicity, coagulopathy is frequently observed within 1 month after CAR-T cell infusion (156, 157). Its severity shows a positive correlation with CRS grade as well as the levels of IL-6 (9), so it’s also known as CRS-related coagulopathy. The recent studies have reported that the incidence of coagulopathy during CAR-T therapy is approximately 50% (9, 156). There are multiple abnormal coagulation parameters in patients with CRS-related coagulopathy, mainly characterized by the elevated levels of D-dimer, the increased fibrinogen degradation products, and the decreased levels of fibrinogen as well as the prolonged prothrombin time. The progress of CRS-related coagulopathy can be divided into three stages, including hypercoagulable stage, consumptive hypo-coagulable stage, and hyperfibrinolysis stage (9). At hypercoagulable stage, the patients mainly present with excessive micro-thrombosis, which can be treated with anticoagulant drugs, such as low-molecular-weight heparin. At consumptive hypo-coagulable stage, the individuals exhibit bleeding, accompanied by the decreased fibrinogen levels and the prolonged APTT and PT, so replacement treatment is required. At hyperfibrinolysis stage, the fibrinogen level is substantially decreased and the level of D-Dimer is significantly up-regulated. Once the fibrinogen level in plasma is lower than 1.5 g/L, the fibrinogen concentrate or cryoprecipitate replacement should be administrated (**Table 2** toxicities related to CAR-T cell therapy**)** Given that cytokine storm plays an essential role in CRS-related coagulopathy, the early and effective management of CRS might be helpful to reduce the incidence of coagulopathy. Without timely and effective intervention, a part of patients with coagulopathy may further develop disseminated intravascular coagulation (DIC), accompanied by the poor prognosis (9, 156, 158).

**Table 2:** Toxicities related to CAR-T cell therapy and effective solutions.

Toxicities	Manifestations	Solutions	Reference
**CRS**	grade 1 CRS: fever, fatigue, myalgia, nausea, and/or malaise	supportive care	(117)
	grade 2 or higher CRS: fever, hypoxia, hypotension, and organ dysfunction	tocilizumab, corticosteroids, and intensive care	
**CRES**	headache, dizziness, delirium, seizures, cerebral edema, and coma	tocilizumab, corticosteroids, anakinra, and intensive care	(93, 95, 117)
**B cell aplasia**	hypogammaglobulinemia	immunoglobulin infusion	(128, 136, 137, 141)
	HBV reactivation	antiviral prophylaxis	
	herpesvirus infections	acyclovir	
**Cytopenia**	anemia	red blood cell transfusions	(154)
	leukopenia	granulocyte colony-stimulating factor, protective isolation	
	thrombocytopenia	platelet transfusions, thrombopoietin, romiplostim	
**CRS-related coagulopathy**	hypercoagulable stage: extensive micro-thrombosis, normal/shortened APTT and PT	anticoagulant treatment: low-molecular weight heparin	(9)
	consumptive hypo-coagulable stage: hemorrhage, decreased fibrinogen levels, and prolonged APTT and PT	anticoagulant treatment combined with replacement treatment: low-molecular weight heparin, fresh-frozen plasma, cryoprecipitate, fibrinogen concentrate	
	Hyperfibrinolysis stage: hemorrhage, hypofibrinogenemia, significantly increased levels of D-Dimer and FDP, and prolonged APTT and PT	replacement treatment and antifibrinolytic treatment: cryoprecipitate, fibrinogen concentrate	

**Figure 4:**
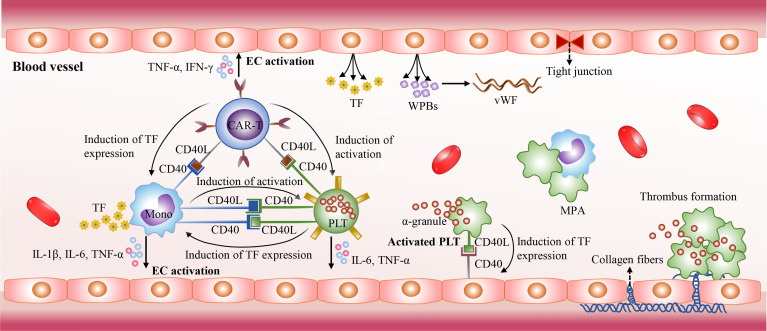
The mechanisms of CRS-related coagulopathy. The CD40/CD40L interactions also play an essential role in CRS-related coagulopathy. The activated CAR-T cells with high CD40L expression mediate platelet activation in a CD40-independent manner. Then the activated platelets express high levels of CD40L. It could stimulate endothelial cell activation, and induce the expression of TF in monocytes and endothelial cells through direct interaction with CD40. Then TF triggers the extrinsic coagulation cascade. In addition, the CD40/CD40L interactions stimulate the excessive release of cytokines, such as IL-1β, TNF-α, and IL-6. High levels of cytokines further mediate endothelial injury and promote the release of TF and Weibel-Palade bodies (WPBs). The WPBs contains von Willebrand factor (VWF) which plays an essential role in platelet adhesion and aggregation. Due to endothelial injury, collagen fibers are exposed and activate intrinsic coagulation pathway.

The mechanisms of CRS-related coagulopathy remain unclear. The activated platelets, monocytes, and endothelial cells as well as the CD40/CD40L interactions between them may collectively contribute to CRS-related coagulopathy. The CD40L expressed on activated CAR-T cells induces platelet activation in a CD40-independent manner in blood circulation (159). The activated platelets are prone to the form monocyte-platelet aggregates (MPAs) and are highly express CD40L, which could induce the expression of tissue factor (TF) in monocytes and endothelial cells through the direct interaction with CD40 (159–162). TF could activate the extrinsic coagulation cascade, and monocytes are the major sources of TF. Besides the CD40/CD40L interactions, there are also a variety of inducers could stimulate monocytes to upregulate the expression of TF, such as C-reactive protein (CRP), IL-1β and TNF-α (163–165). In addition, the CD40/CD40L interactions between them promote the excessive production of cytokines as well, such as IL-1β, TNF-α, and IL-6. High levels of cytokines further mediate endothelial injury and result in the release of TF, the procoagulant particles Weibel-Palade bodies (WPBs), and von Willebrand factor (VWF) as well as the exposure of the collagen fibers. The exposed collagen fibers trigger intrinsic coagulation pathway. In addition, cytokines IL-6, TNF-*α*, and IFN-*γ* can directly inhibit the production and activity of ADAMTS13, which contributes to the elevated levels VWF in blood and promote platelet adhesion and aggregation (166, 167). Moreover, serious liver damage induced by CAR-T cell therapy influences the production of coagulation factors, and some patients with hematological malignancies had be in a hypercoagulable state prior to CAR-T cell therapy (168) (**Figure 4**).

## Resistance to CAR-T Cell Therapy and Potential Effective Strategies

However, with the widespread application of CAR-T cell therapy in R/R B-cell malignancies, a considerable proportion of patients relapse after CAR-T cell therapy. There are multiple factors which contribute to relapse after CAR-T cell therapy, including antigen escape, the limited CAR-T cell persistence, and immunosuppressive tumor microenvironment. There are some therapeutic strategies to overcoming the resistance to CAR-T cell therapy, including the application of bispecific or armored CAR-T cells, optimizing the CAR structure, combining CAR-T cell therapy with other approaches, such as small‐molecule drugs, localized radiotherapy, and oncolytic viruses.

### Overcoming Antigen Escape

Although CAR-T cell therapy has made impressive achievements in R/R B cell malignancies, a majority of patients still relapse (143). One of the primary mechanisms of relapse after CAR-T cell therapy is antigen loss (169, 170). The antigen mutations under therapeutic pressure of CAR-T cell therapy are the most common mechanisms of antigen loss, including splice variants, lineage switching, and biallelic mutations (171–173). In addition to antigen mutations, the lower antigen density on the surface of induced by the endocytosis of CAR-T cells could promote tumor immune escape as well (174). Last but not least, a study has reported that the anti-CD19 CAR was incidentally transferred into a leukemic B cell during CAR-T cell manufacturing and then bound to the CD19 epitope on leukemic blasts, so “epitope-masking” prevented leukemic blasts from being recognized by anti-CD19 CAR-T cells (175).

Targeting distinct antigens is one of the most effective approaches to overcome antigen-negative relapse. The dual-targeting CAR-T cells which could recognize two distinct target antigens have been demonstrated to reduce the risk of antigen-negative relapse, such as the bispecific CAR-T cells in B cell lymphoma/leukemia and the APRIL-based CAR-T cells targeting both BCMA and TACI in MM (86, 176–180). The multi-targeted CAR-T cells have also been explored. It has been demonstrated that the tri-specific CD19-CD20-CD22-targeting CAR-T cells could rapidly eliminate B cell lymphoma in a preclinical study (181), and BAFF ligand-based CAR-T cells simultaneously target three receptors, including BAFF-R, BCMA, and TACI (182). In addition to simultaneously targeting different antigens, increasing immunogenicity of target cells might be a feasible strategy. For example, small molecule γ-secretase inhibitors could reduce the shedding of BCMA and promote the recognition of MM cells by CAR-T cells (183).

The γδ T cells (γδ T) are a small population of peripheral blood cytotoxic T cells, which express both T cell receptors (TCRs) and natural killer receptors (NKRs), and involved in anti-tumor immunity. In particular, NKRs expressed on γδ T cells play a major role in tumor cell recognition in hematological malignancies (184–186). Thus, besides antigen recognition mediated by the scFvs, γδ CAR-T cells could also recognize antigen-negative leukemia cells *via* NKRs in an MHC-independent manner (187). Moreover, γδ T cells did not induce graft-versus-host disease (GVHD) in allogeneic and HLA-haploidentical hematopoietic stem cell transplantation, which indicates that γδ T cells don’t trigger alloreactivity (188, 189). Thus, they are more suitable for the development of universal CAR-T cells.

### Regulating the Persistence of CAR-T Cells

The short-term persistence of CAR-T cells limits their anti-tumor efficacy and may result in antigen-positive relapse. There are multiple strategies to improve the persistence of CAR-T cells, such as optimizing CAR-T cell construct, utilizing memory T cells, and rationally designing the ratio of CD4 to CD8 CAR-T cells (190). To date, CD28 and 4-1BB are the most common co-stimulatory molecules in CAR-T cell products. However, it has been demonstrated that 4-1BB co-stimulation could ameliorate CAR-T cell exhaustion compared with CD28 co-stimulation (191, 192). Remarkably, combining CD28 and 4-1BB could simultaneously augment the anti-tumor effects and increase the persistence of CAR-T cells (193–196). In addition, the CAR-T cell structure can be optimized by the fully humanized CARs. The humanized CAR-T cells could avoid the rejection by the host immune system, and they were still effective in R/R patients who have failed in prior murine CAR-T cell therapy (68, 197). CD4^+^ T cells exhibit developmental plasticity and can directly kill tumor cells (198), but they eliminate tumor cells at the slower rate and release the lower levels of Granzyme B than CD8^+^ T cells. Thus, CD4^+^ CAR-T cells exhibit a superior persistence (199–202), and the ratio of CD4/CD8 CAR-T cells may influence the therapeutic efficacy. Currently, CD4/CD8 CAR-T cells at a 1:1 ratio have been demonstrated to exert excellent anti-tumor effects (200).

CAR-T cells have been considered as “living drugs”, but they lack the precise regulation. Given that the excessive expansion of CAR-T cells could lead to the life-threatening CRS, so it is necessary to regulate the expansion and persistence of CAR-T cells to mitigate unexpected or severe toxicities through the addition of the safety switches. The well-known inducible caspase 9 (iCasp9) suicide gene and the small molecule control systems have been explored (203, 204). In small molecule control systems, the FDA-approved small molecule drugs act as the key switches to specifically regulate antigen recognition or deplete CAR-T cells, such as lenalidomide, methotrexate, alemtuzumab, rituximab, and cetuximab (31, 63, 205–208), as well as orthogonal IL-2 (205, 206). The above-mentioned monoclonal antibodies mediate the depletion of CAR-T cells through antibody-dependent cell-mediated cytotoxicity (ADCC) or complement-dependent cytotoxicity (CDC) (63, 209). Nevertheless, the depletion of CAR-T cells is slow in this strategy, which may be not suitable for patients with severe toxicities.

### Improving Anti-Tumor Efficacy of CAR-T Cell Therapy Through Combinatorial Strategies

The low activity of CAR-T cells could also limit the efficacy of CAR-T cell therapy. Multiple immune-stimulatory molecules, including certain cytokines or co-stimulatory molecules, have been demonstrated to play an important role in regulating the development and function of T cells, such as IL-7, IL-12, IL-15, IL-18, IL-21, and CD40L (109, 210–214). They could promote the robust expansion of the CAR-T cells and increase memory-phenotype CAR-T cells as well as improving their persistence (215–218). In addition to adding these exogenous cytokines, the genetic modifications to constitutively express these immune-stimulatory molecules or their receptors could also improve the function of CAR-T cells (10, 213, 219, 220). These fourth-generation CAR-T cells have showed the improved anti-tumor activities by stimulating the activation of themselves or endogenous immune cells in a autocrine or paracrine manner (11, 220–222).

To improve the efficacy of CAR-T cell therapy, combining CAR-T cell therapy with small‐molecule drugs appears to be promising and may produce synergistic effects. The selective inhibitors of nuclear export selinexor, lenalidomide and carfilzomib have been approved for the treatment of MM (223, 224). Intriguingly, combining them with CAR-T cells also achieved encouraging outcomes, with the improved cytotoxic activity and cytokine production of CAR-T cells (225, 226). In particular, the recent clinical studies showed that the R/R MM patients resistant to anti-BCMA CAR-T cell therapy could also benefit from selinexor-based regimens and carfilzomib-based regimens (227, 228), and a study reported that anti-BCMA CAR-T cell therapy combined with lenalidomide was effective in the R/R MM patients who had previously relapsed after anti-BCMA CAR-T cell therapy (229). Ibrutinib, a well-known Bruton’s tyrosine kinase inhibitor, has been approved for the treatment of CLL and MCL. Importantly, Ibrutinib not only improved CAR T cell-anti-tumor efficacy in both preclinical and clinical studies, but also reduced the risk of severe CRS (230–233). In addition, it has been demonstrated that demethylating agents azacitidine and decitabine could enhance cytotoxic effect of CAR-T cells as well (234–236). Besides, CAR-T cell therapy in combination with inhibitors of antiapoptotic proteins could overcome the resistance induced by antiapoptotic proteins (237). However, CAR-T cell therapy combined with small‐molecule drugs is still in its infancy, and numerous combinational strategies are being explored.

Additionally, the localized radiotherapy could serve as a well-tolerated and effective bridging strategy between the leukapheresis and CAR-T cell infusion for lymphoma or MM patients with bulky disease (238–240). On the one hand, this combinational therapy could prevent disease progression and reduce tumor burden; On the other hand, it may sensitize the CAR-T cells through the abscopal effect, which may be associated with the upregulation of intratumoral chemokines and cytokines, the release of neo-antigens, and the activation of endogenous immune cells (241–245). Nonetheless, the optimal irradiation dose and fractionation remain to be identified.

### Overcoming Immunosuppressive Microenvironment

While directly targeting tumor cells is important, it is also critical to overcome the immunosuppressive tumor microenvironment. Although tumor microenvironment is believed to play a relatively minor role in drug resistance in hematological malignancies, MM, leukemia, and lymphoma microenvironment also contains tumor supportive components, such as stromal cells, myeloid-derived suppressor cells, regulatory T-cells, tumor-associated macrophages, and tumor-associated neutrophils (246–250), which interact closely with malignant cells and promote their survival as well as immune escape (250–253). In addition, these immunosuppressive components impair the cytotoxic effects of CAR-T cells and result in CAR-T cell exhaustion (248, 249). Therefore, it is also necessary to overcome the immunosuppressive microenvironment in hematological malignancies. In addition to armored CAR-T cells, CAR-T cell therapy in combination with checkpoint blockades or oncolytic viruses also appears to be an appealing strategy.

The PD-1/PD-L1 pathway plays a major role in T cell exhaustion and represents a major mechanism of tumor immune escape. Thus, blockage of PD-1/PD-L1 interaction could promote the immune system to fight against cancer cells, and PD-1 blockade has achieved tremendous success in diverse tumor types in recent years, especially in lymphoma (254, 255). PD-1 blockade is usually administrated in combination with conventional chemotherapy or other immunotherapies. Currently, multiple studies have explored the combination therapy with CAR-T cells and PD-1 blockade (**Table 3** combinatorial strategies with CAR-T cell therapy). In a clinical trial enrolled 11 NHL patients, 45.5% of patients achieved CRs after this combinatorial therapy, and the toxicities were well-tolerable (256, 257). The mechanisms may be mainly attributed to the decreased CAR-T cell exhaustion (258–260). In addition, the CAR-T cells which could secrete the PD-1 blocking scFv have been developed. The preclinical study demonstrated that the efficacy of such CAR-T cells was equally effective or superior to the combinational therapy of CAR-T cells and PD-1 inhibitor (261). TIM-3 is another inhibitory immune checkpoint, and the combination of TIM-3 blockade with CAR-T cells exerts synergistic anti-tumor activity as well (262).

**Table 3:** Combinatorial strategies with CAR-T cell therapy reported in clinical studies.

Combinatorial approach	Disease	Target	Reference
**PD-1 blockade**	R/R NHL	CD19	(256, 257)
**Ibrutinib**	R/R CLL, MCL, FL	CD19	(230, 231, 233)
**Radiotherapy**	R/R NHL	CD19	(238–241)
**Decitabine**	R/R NHL	CD19	(236)
**Lenalidomide**	R/R MM	BCMA	(229)
**Selinexor-based regimens**	R/R MM	BCMA	(228)
**Carfilzomib-based regimens**	R/R MM	BCMA	(227)

The combination of CAR-T cell therapy and oncolytic viruses is an innovative strategy to overcome immunosuppressive microenvironment. The virus-infected tumor cells which carry pathogen-associated molecular patterns (PAMPs) could recruit host immune cells and thereby promote the recognition of TAAs by the host immune system and the oncolytic viruses also can be genetically modified with immune-stimulatory molecules to enhance the anti-tumor activity of CAR-T cells (263, 264). Besides, oncolytic viruses directly lyse tumor cells and result in the release of TAAs and damage-associated molecular patterns (DAMPs), which could increase tumor immunogenicity and activate APCs through pattern recognition receptors (PRRs), eventually activating tumor-specific T cells (265–267).

In addition, the armored CAR-T cells which express the immune-regulatory molecules, such as IL-15, IL-18, CD40L as well as TGF-β dominant-negative receptor II, are able to remodel the tumor microenvironment (211, 219, 220, 268). Oncometabolites in the tumor microenvironment could inhibit the metabolism and function of CAR-T cells, so suppressing the accumulation oncometabolites is a potential therapeutic option. Kynurenine (Kyn) is an oncometabolite which exists in various hematopoietic malignancies, such as lymphoma and leukemia, and the enzyme kynurenine catalyzes the degradation of Kyn. Thus, the anti-CD19 CAR-T cells were genetically modified with the enzyme kynurenine gene, and they exhibited the improved anti-tumor activity against Nalm6-GL cells in the immunosuppressive tumor microenvironment with high Kyn (269). Interestingly, some target antigens are co-expressed on immunosuppressive cells in tumor microenvironment, so targeting these antigens can simultaneously eliminate malignant cells and immunosuppressive cells. For example, anti-CD123 CAR-T cells target both malignant cells and TAMs in HL (270).

## Conclusions

There are many challenges and opportunities presented by CAR-T cell therapy. With the identification of novel therapeutic targets and the optimization of CAR constructs, CAR-T cell therapy will have broader clinical applications, beyond hematological malignancies. However, with the rapid commercialization of CAR-T cell therapy, it poses a significant challenge for the management of CAR-T cell therapy, such as the toxicities associated with CAR-T therapy and relapse after CAR T-cell therapy. Therefore, exploring their underlying mechanisms and overcoming these limitations will help R/R patients gain more benefits from this promising. Currently, multiple combinatorial approaches with CAR-T cell therapy are being explored and seem to be promising immunotherapy. In addition, UCAR-T cells and CAR-NK cells also show great potential in cancer treatment due to their low manufacturing costs and off-the-shelf availability.

## Author Contributions

YX, XZ, and LZ designed the manuscript. XZ, HZ, and SC drafted the manuscript and created the figures. XZ and YX revised the manuscript. All authors read and approved the final manuscript.

## Funding

This work was supported by the National Natural Science Foundation of China (Grant No. 81973583 and 81873426), and Guangdong Basic and Applied Basic Research Foundation (2021A1515011575).

## Conflict of Interest

The authors declare that the research was conducted in the absence of any commercial or financial relationships that could be construed as a potential conflict of interest.

## Publisher’s Note

All claims expressed in this article are solely those of the authors and do not necessarily represent those of their affiliated organizations, or those of the publisher, the editors and the reviewers. Any product that may be evaluated in this article, or claim that may be made by its manufacturer, is not guaranteed or endorsed by the publisher.
